# A Novel Synthetic Method for N Doped TiO_2_ Nanoparticles Through Plasma-Assisted Electrolysis and Photocatalytic Activity in the Visible Region

**DOI:** 10.3389/fchem.2018.00458

**Published:** 2018-10-02

**Authors:** Tae Hyung Kim, Gwang-Myeong Go, Hong-Baek Cho, Yoseb Song, Chan-Gi Lee, Yong-Ho Choa

**Affiliations:** ^1^Advanced Materials and Processing Center, Institute for Advanced Engineering (IAE), Yongin, South Korea; ^2^Department of Fusion Chemical Engineering, Hanyang University, Ansan, South Korea

**Keywords:** plasma enhanced electrolysis, amorphous N-doped TiO_2_, visible region photocatalyst, metal-direct synthesis, nanoparticle synthesis

## Abstract

Nitrogen doped TiO_2_ (N-TiO_2_) nanoparticles were synthesized via a novel plasma enhanced electrolysis method using bulk titanium (Ti) as a source material and nitric acid as the nitrogen dopant. This method possesses remarkable merits with regard to the direct-metal synthesis of nanoparticles with its one-step process, eco-friendliness, and its ability to be mass produced. The nanoparticles were synthesized from bulk Ti metal and dipped in 5–15 mmol of a nitric acid electrolyte under the application of AC 500 V, the minimum range of voltage to generate plasma. By controlling the electrolyte concentration, the nanoparticle size distribution could be tuned between 12.1 and 24.7 nm using repulsion forces via variations in pH. The prepared N-TiO_2_ nanoparticles were calcined at between 100 and 300°C to determine their photocatalytic efficiency within the visible-light region, which depended on their crystal structure and N doping content. Analysis showed that the temperature treatment yielded an anatase TiO_2_ crystalline structure when the N doping content was varied from 0.4 to 0.54 at.%. In particular, the 0.4 at.% N doped TiO_2_ catalyst exhibited the highest catalytic performance with quadruple efficiency compared to the P-25 standard TiO_2_ nanoparticles, which featured a 91% degradation of methyl orange organic dye within 300 min. This solid-liquid reaction based on plasma enhanced electrolysis could open new pathways with regard to high purity mass producible ceramic nanoparticles with advanced properties.

## Introduction

Intensive research has been performed to produce highly photocatalytic titanium dioxide (TiO_2_) nanoparticles (Ba-abbad et al., 2012; Mattioli et al., 2014; Vukoje et al., 2016) for a large application space, including water splitting (Galinska and Walendziewski, 2005; Ni et al., 2007; Tang et al., 2008) and industrial pollutant degradation (Liu et al., 2010; Lin et al., 2012; Dong et al., 2015). However, crystalline TiO_2_ nanoparticles possess limited photocatalytic activity due to their wide band gap regardless of crystal structure from anatase to rutile (anatase ~ 3.2 eV, rutile ~ 3.0 eV); the nanoparticles only absorb within the UV region, comprising a small portion of sunlight of <5% of the solar flux. Thus, to efficiently enhance the photocatalytic activity, many researchers have focused on utilizing the visible region, which contains nearly half of the energy from sunlight (~45%) by doping metal cations (Ag, C, Zn, etc.) into the Ti lattice (Cao et al., 2008; Aware and Jadhav, 2015). However, cation dopants located at d-states within the band gap act as trap centers for excited electron and holes, which can additionally shift the conduction band below the redox potential (Wu et al., 2007). Thus, some drawbacks exist such as a low thermal stability and can act as recombination centers in the form of charge carriers. Alternatively, anion dopants such as N, S, and P have attracted considerable attention due to their intrinsic properties, which possess similar characteristics to oxygen. Among those anions, nitrogen appears to be the most plausible ingredient to be investigated for doping with the formation of a metastable center, low ionization energy state, and reduced atomic size (Dunnill et al., 2011). The light absorption range can thus be extended into the visible region (up to 380 nm) through existing p-states near the valance band (Scanlon et al., 2013).

The preparation of N-TiO_2_ nanoparticles has been extensively studied with aqueous-based chemical processes such as sol-gel (Yu et al., 2007; Dunnill et al., 2011; Caratto et al., 2012), hydrothermal (Zhou et al., 2011), solvothermal (Li and Liu, 2009; Yang et al., 2010), and polymeric precursor methods (Soares et al., 2011; Yu et al., 2017) being the most frequently employed. These are general methods for the preparation of nanoparticles that can be used to control morphology, structure, and particle size. However, synthetic conditions such as the solution temperature, pH, reaction time, and precursor ratio must be sensitively controlled. Moreover, long reaction times and drying processes are essential in order to obtain highly pure and crystalline nanoparticles, making mass production difficult. To overcome these drawbacks, a noble synthetic strategy using plasma enhanced electrolysis was adopted herein. The process utilizes a small amount of electrolyte, characterizes a quick production time, and entails the direct synthesis of nanoparticles from bulk metal in a manner suitable for mass production. Furthermore, this technology is environmental friendly due to employing a reusable electrolyte and not requiring an additional nitrogen source such as ammonia (Gao et al., 2014).

Here we report a novel method for the preparation of interstitial N-TiO_2_ nanoparticles by bulk-direct synthesis using an eco-friendly and mass producible method via plasma enhanced electrolysis, enhancing photocatalytic activity through the narrowing of band gap structures, which will widen the light absorption region into the visible spectrum. During the nanoparticle synthetic process, arc plasma was formed on the metal surface, creating gaseous anionic species that subsequently reacted with cationic Ti species to grow into N-TiO_2_ through nucleation. The relationship between N doping content and nanoparticle crystallinity, which primarily affected the photocatalytic efficiency was analyzed and elucidated by comparing samples prepared through the variation of electrolyte concentrations and calcination temperatures.

## Materials and methods

### Synthesis of amorphous N-TiO_2_ nanoparticles

**Figure 1:**
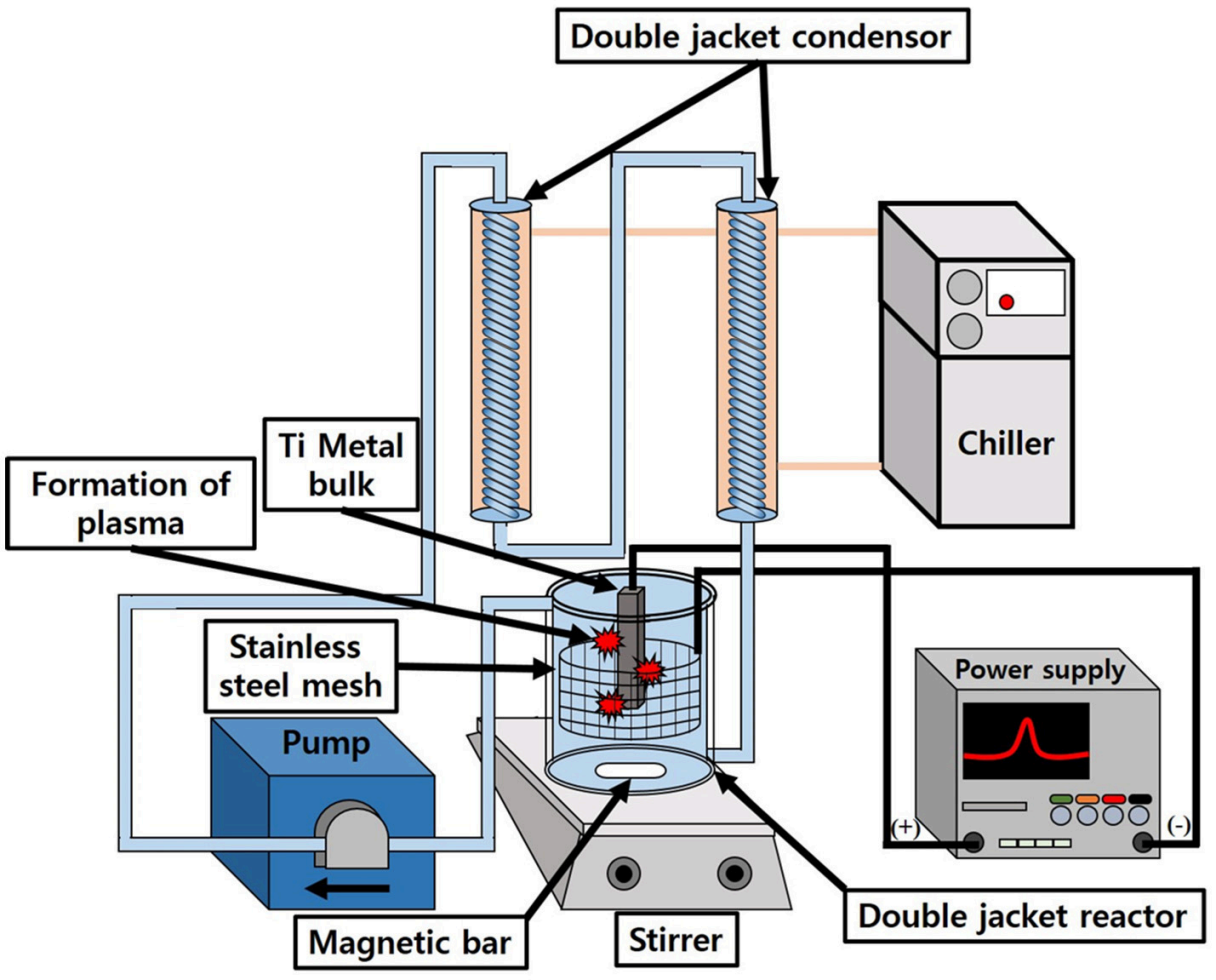
Schematic diagram for liquid-based plasma enhanced electrolysis.

Figure 1 shows a brief schematic diagram for the synthesis of N-TiO_2_ nanoparticles by the generation of plasma. A Ti metal plate (99.99% purity) was first washed twice with acetone and deionized water in order to remove other possible impurities. The organic-free Ti plate was hanged in 1 L of a nitric acid electrolyte in the middle of a double-jacket reactor. The Ti metal was connected to an anode and stainless steel mesh (SUS 304, 60 mesh) with a cylindrical shape that was connected to the cathode; the shape of the cathode was designed to radially impart an equal supply of electric voltage to the metal surface. The solution temperature was controlled using a bath circulator and stirred with a magnetic bar to exchange heat efficiently under the high temperature plasma reaction. After the initial generation of plasma on the metal surface, the voltage and current density of the power supply was maintained at certain conditions such as AC 500 V at 100 Hz for 10 min to synthesize nanoparticles continuously; the nitric acid concentration was varied from 5 to 15 mmol to observe the effect of the electrolyte concentration on the morphology of TiO_2_. After the reaction, the nanoparticle suspension was centrifuged to remove the remaining electrolyte and dried in a vacuum oven at 80°C for 24 h. The as-synthesized powders were calcined at different temperatures: 100, 150, 200, 250, and 300°C to transform amorphous TiO_2_ into the crystalline anatase phase to investigate their respective photocatalytic activity.

### Sample characterization

Scanning electron microscopy (SEM) images were obtained with a Hitachi S-4800/Horiba EX-250 to analyze the morphology of the synthesized N-TiO_2_ nanoparticles. High-resolution transmission electron microscopy (HR-TEM) images were obtained with a JEOL, JEM-2100F to measure and calculate lattice parameters with selected area electron diffraction (SAED) patterns. The nanoparticle crystallinity and structures were analyzed with a RigaKu D/MAX-2500/PC X-ray diffractometer (XRD) using Cu Kα_1_ radiation (λ = 1.54 nm 2θ range between 20 and 80°). X-ray photoelectron spectroscopy (XPS) was performed with a Thermofisher Scientific K-Alpha+ MXP10. The photocatalytic activity of the N-TiO_2_ nanoparticles was assessed by analyzing the visible region efficiency of the decomposing methyl orange (MO, Sigma-Aldrich Co.) solution in a dark chamber equipped with a UV cut-off filter (Edmund Optics, 400–50 mm Diameter, OD 2 long pass filter) and a 300 W Zenon lamp. While being exposed to visible light, degraded methyl orange was collected with nanoparticles at 2 h intervals and separated via centrifugation. After separation, a Dong-il Shimadzu Co. UV-2600 UV-vis spectrophotometer was employed to calculate the photocatalytic activity. To compare the recombination rates of the samples, photoluminescence (PL) measurements were performed using a 290 nm laser (Horiba, Nanolog) as the excitation source at room temperature.

### Photocatalytic activity analysis

The photocatalytic efficiency of the N-TiO_2_ samples was compared after various degrees of heat treatment: 100, 150, 200, 250, and 300°C for 4 h to transform the crystal structure and yield variations with regard to the degree of N doping with Degussa P-25 standard TiO_2_ nanoparticles. After calcination, 100 mg of nanoparticles were added and mixed with 100 ml of a 1 × 10^−5^ mol/L MO organic dye solution to measure the decomposition efficiency of each sample. The 100 mg/L powder mixed MO solution was sonicated for 10 min prior to the degradation step to enhance solution dispersity. The well-dispersed organic solution was placed in a dark chamber and stirred for an additional 30 min for stability. The degradation process was performed in a double-jacketed reactor to control the solution temperature and was irradiated with light using a 300 W Xe lamp with a UV cut-off filter. The reaction solution was collected every 2 h and analyzed via UV-vis spectroscopy to calculate the degradation efficiency for a 6 h long reaction.

## Results and discussion

### Synthesis of N-TiO_2_ nanoparticles

**Figure 2:**
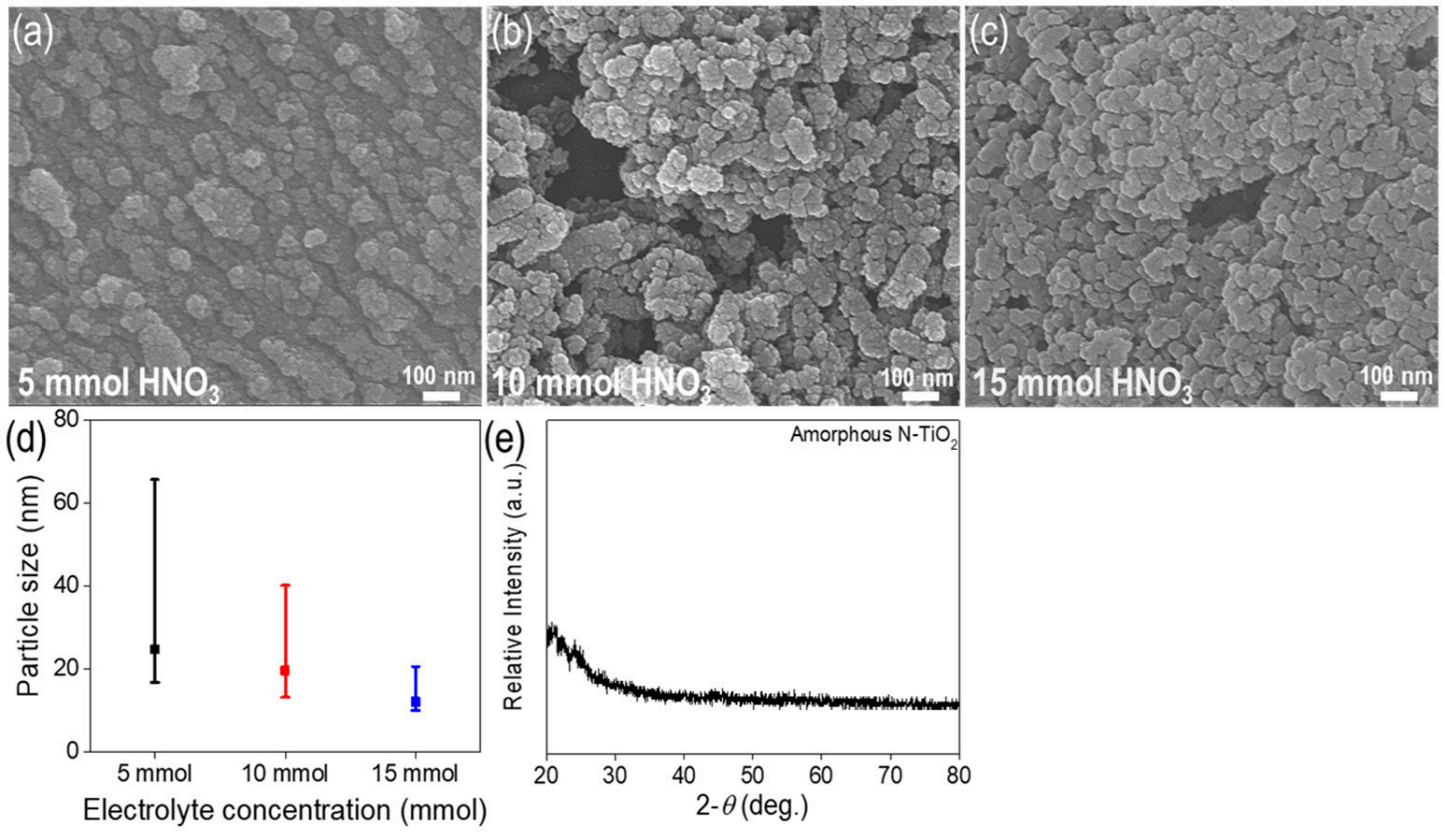
Characterization of N-TiO_2_ nanoparticles with various electrolyte concentrations,SEM images of **(a)** 5 mmol, **(b)** 10 mmol, **(c)** 15 mmol, **(d)** the size distribution and **(e)** XRD data.

Figures 2a–c show the TiO_2_ nanoparticle morphologies produced at different concentrations of nitric acid. The particle size distribution analyzed via SEM decreased between the range of 12.1–24.7 nm as the nitric acid concentration was increased (size distribution in Figure 2d). The mean particle size decreased to 24.7, 19.4, and 12.1 nm at concentrations of 5, 10, and 15 mmol, respectively. It could be seen that at lower concentrations, some rod-like nanoparticle shapes could be found. Irregular shaped nanoparticles indicated unbalanced nanoparticle synthesis between the nucleation and growth steps. At higher concentrations with a lower pH electrolyte, more uniform and non-agglomerated nanoparticles could be produced. This could be attributed to two factors; repulsion forces and balance between the nucleation and growth steps (Alqadi et al., 2014). When the electrolyte solution pH decreased to lower values, ions such as OH^−^, which reacted with metal ions, scarcely existed and facilitated a slower nucleation and growth process. This also increased repulsion forces between nanoparticles, thus producing much more uniform nanoparticles with a smaller size distribution than at higher pH values (Wu et al., 2011). At higher electrolyte concentrations, the solution conductivity increased due to a larger quantity of ionized ions such as H^+^ and SO${}_{4}^{-}$ (when H_2_SO_4_ was used as an electrolyte), yielding a higher current density that was related to the plasma intensity; this would produce transparent solutions without forming colloid nanoparticles, thus necessitating the use of additional reducing agents (Kim et al., 2016). As a result, 15 mmol was the optimum nitric acid concentration for nanoparticle synthesis.

Figures 2d,e show size distribution and XRD data for the synthesized amorphous N-TiO_2_ nanoparticles. The size distribution showed the nanoparticles ranging from 12.1 to 24.7 nm (Figure 2d); the size deviation increased noticeably from ±4.7 to ±22.2 nm as the electrolyte concentration increased. A broad peak at the lower degree with no other specific peaks in the XRD data (Figure 2e) indicated the pristine amorphous structure of N-TiO_2_. Moreover, no peaks for the Ti bulk metal starting material were detected, implying that Ti was completely transformed to TiO_2_ nanoparticles by adopting the plasma enhanced electrolysis process. If detached from the metal surface, the particle peaks would exhibit XRD peaks for bulk Ti and several irregularly shaped particles.

**Figure 3:**
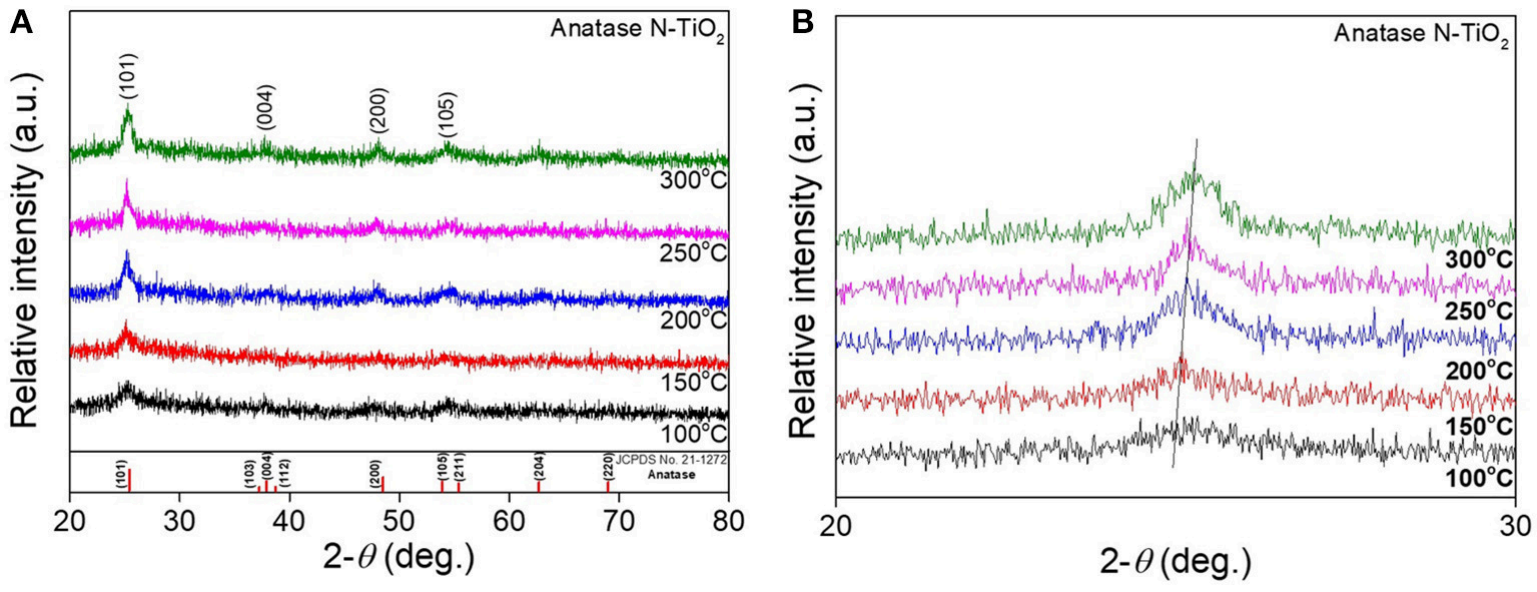
XRD analysis of **(A)** N-TiO_2_ nanoparticles at different calcination temperature and **(B)** an enlarged (101) peak.

After the synthesis of nanoparticles using a 15 mmol electrolyte concentration, amorphous N-TiO_2_ nanoparticles were calcined at various temperatures to compare the XRD peaks between the treated nanoparticles in Figure 3. The as-prepared amorphous N-TiO_2_ nanoparticles revealed that the amorphous phase was transformed to a metastable anatase structure after being calcined at 100, 150, 200, 250, and 300°C for 4 h. In Figure 3A, analyzed peaks for the (101), (004), (200), and (105) lattice planes matched those for anatase TiO_2_ (JCPDS card no. 21-1272) and no nitrogen source peaks were detected, which indicated that nitrogen was perfectly doped into the TiO_2_ lattice (as confirmed in Figure 6). With an increase in calcination temperature, the XRD peak intensity also increased, indicating that the calcination temperature enhanced the crystallinity of the N-TiO_2_ nanoparticles with a deformation of amorphous bonding between atoms during calcination. Additional evidence for nitrogen doping was observed by a shift in the enlarged peak of the (101) lattice in Figure 3B. Due to the larger atomic radius of nitrogen compared to oxygen (N^3−^ = 171 Ã > O^2−^ = 140 Ã), a large degree of nitrogen doping would lead to XRD peak broadening effects. A reduction in doping with an increase in the calcination temperature could shift the peak to much higher diffraction angles compared to non-doped TiO_2_ (Sun et al., 2016). This result will be compared and identified via XPS and PL analysis in later sections.

**Figure 4:**
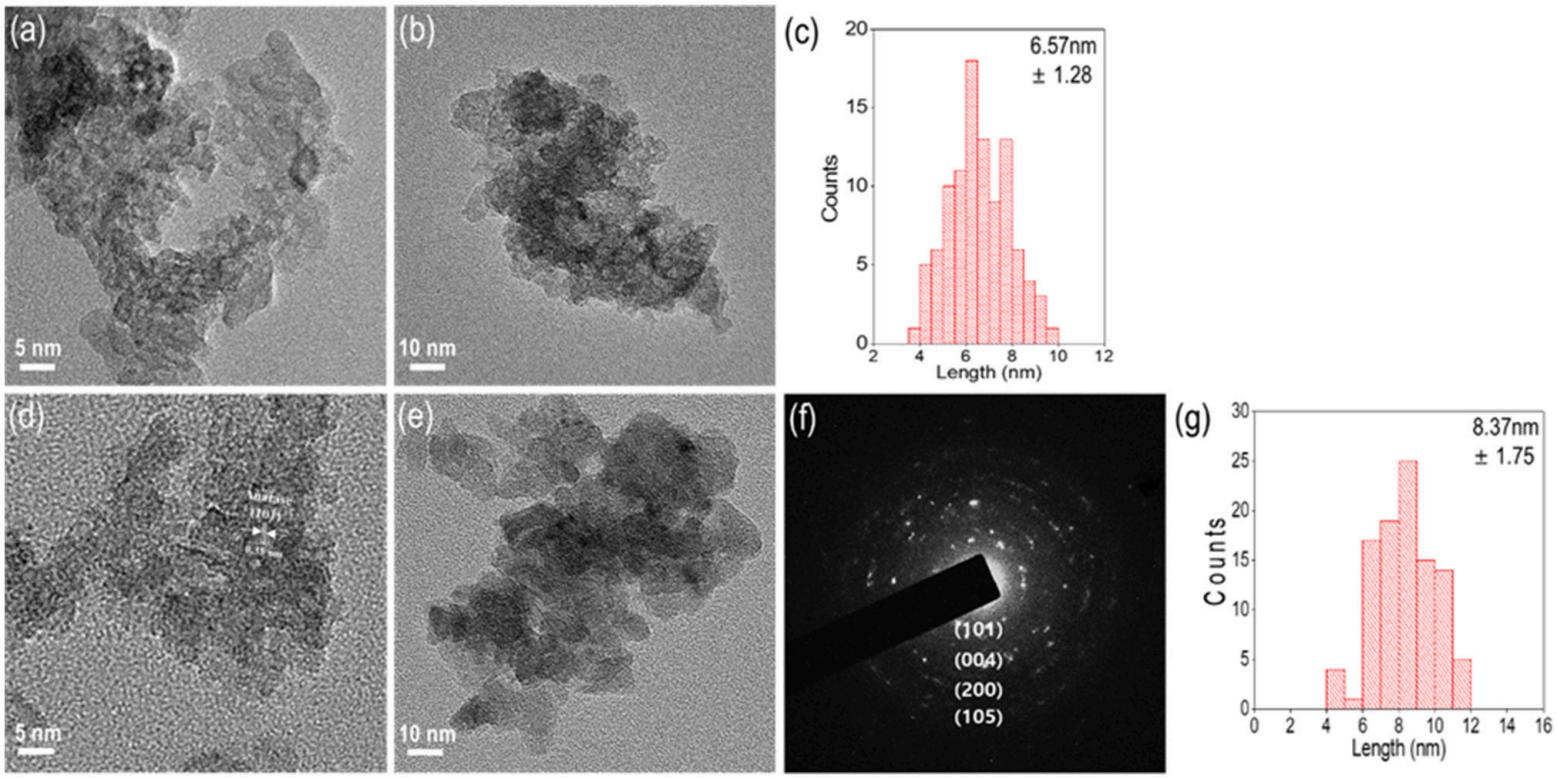
TEM images of an N-TiO_2_ nanoparticle synthesized at 15 mmol electrolyte under **(a)** amorphous high resolution, **(b)** low resolution, **(c)** size distribution, **(d)** 200°C calcined anatase high resolution, **(e)** low resolution, **(f)** anatase SAED pattern, and **(g)** the size distribution.

To analyze a non-agglomerated single nanoparticle, a well-dispersed colloid solution was loaded onto a Cu grid to perform TEM analysis. According to the TEM images of amorphous N-TiO_2_ structures in Figures 4a,b, there were no signs of lattice fringes in the nanoparticles that matched the XRD analysis in Figure 2. The primary particle size distribution of amorphous N-TiO_2_ in Figure 4c revealed a primary mean particle size of 6.57 nm, which was smaller than that in the SEM images mentioned previously (in Figure 2). After calcination at 200°C, the polycrystalline structure with a ring SAED pattern identified the anatase phase of N-TiO_2_ with a lattice plane of (101), (004), (200), and (105) in Figures 4d–f. The mean primary particle size was 8.37 nm, indicating a minor growth in morphology after calcination (Figure 4g).

### XPS analysis of N-TiO_2_ nanoparticles from different calcination temperatures

**Figure 5:**
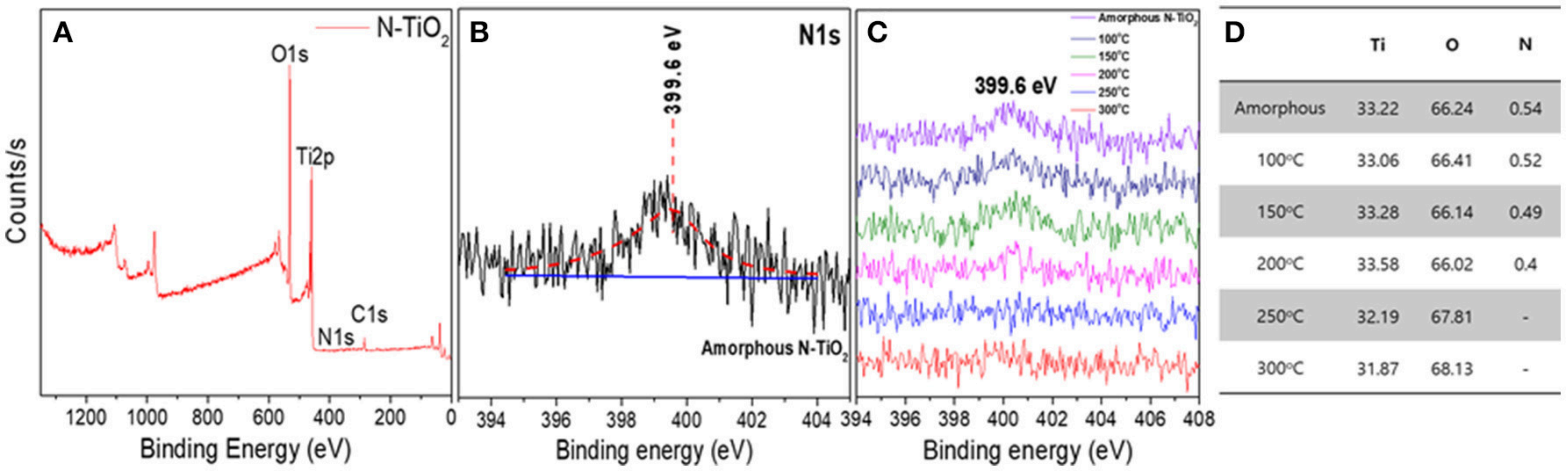
**(A)** XPS analysis of amorphous N-TiO_2_ showing **(B)** an enlarged N1s peak, **(C)** N1s peak at various calcination temperature, and **(D)** the elemental content.

The N doping concentration in the TiO_2_ lattice primarily influenced the electronic structure according to the doping position and state. Thus, synthesized N-TiO_2_ nanoparticles at different calcination temperatures were analyzed via XPS to compare the dopant tendency, as can be seen in Figure 5. The 1s peak located at 284.8 eV was used to calculate the peak shift. The peak at 398.7 eV in Figures 5A,B was evidence of the replacement of N dopants instead of O sites with N-Ti-O bonds (Mohamed et al., 2015). When the N 1s state at 399.6 eV, which was identical and compared to the interstitial doping state with N-Ti-O bonds (Figure 5C), it could be confirmed that the N concentration decreased dramatically from 0.54 to 0.4 at.% by increasing the calcination temperature and lowering the intensity of the N peak, as summarized in Figure 5D, which was consistent with that reported by Cheng et al. (2012). To discuss the interstitial and substitutional N doping states in TiO_2_ lattices, the energy conservation law equation was adapted:

(1) $$\begin{array}{r} E_{f}=E_{TiO_{2}:N}-E_{TiO_{2}}-\frac{m}{2}E_{N_{2}}+\frac{n}{2}E_{O_{2}}, \end{array}$$

where *E*_*Ti*_*O*__2:*N*__ and *E*_*Ti*_*O*__2__ are the total energies for N-doped TiO_2_ and pure TiO_2_, *E*_*N*_2__ and *E*_*O*_2__ indicate the gas energies of N_2_ and O_2_ molecules, and *m* and *n* are integers of doped N atoms and substituted O atoms. According to this theory, energies tended to increase when the N doping level increased by acting as a formed impurity, making it more difficult to achieve a higher doping condition. Also, doping with N atoms in the lattice instead of O atoms required greater bonding energies; interstitial doping was more generally preferable (Zhao and Liu, 2008). In this scenario, the plasma enhanced electrolysis reaction using 5–15 mmol nitric acid could only yield interstitial N doped TiO_2_ nanoparticles.

### Band gap analysis depending on N content

**Figure 6:**
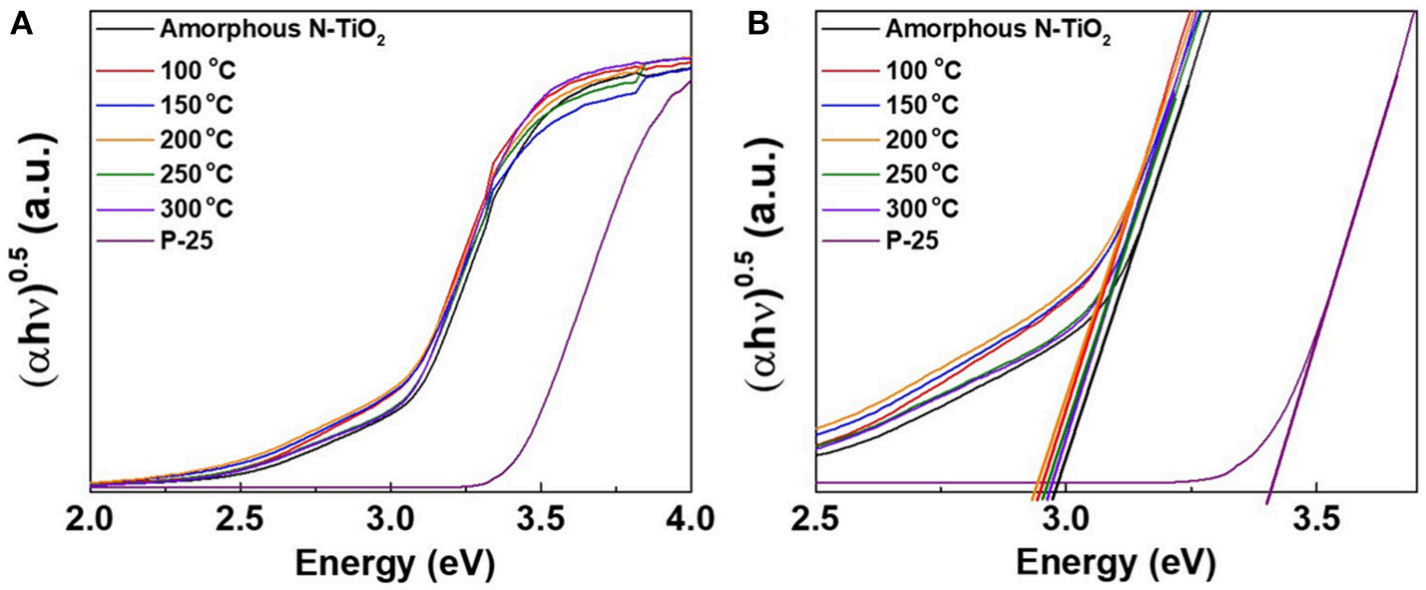
**(A)** Band gap analysis using UV-vis spectroscopy at different calcination temperature and **(B)** enlarged band gap peaks.

Figure 6 and Figure S1 shows the diffuse reflectance spectra of Degussa P-25, amorphous, and various N-TiO_2_ nanoparticle samples calcined at 100, 150, 200, 250, and 300°C, which possess various N doping molar ratios. The peaks of each sample showed absorptions between 300 and 400 nm in Figure 6A and the enlarged peaks in Figure 6B, which exhibited a calculated band gap of 3.4, 3.0, 2.96, 2.94, 2.94, 2.97, and 2.98 eV, respectively. The band gap values exhibited a tendency to red shift toward lower wavelengths with an increase in N doping content that strongly affected the absorption region. Thus, it could be anticipated that nitrogen sources from nitric acid reacted with Ti ions during plasma generation in a form of interstitial doping and modifying the energy state of the TiO_2_ nanoparticle band gap, which could be reduced toward the visible region.

### Photocatalytic activity depending on N doping content and crystallinity

**Figure 7:**
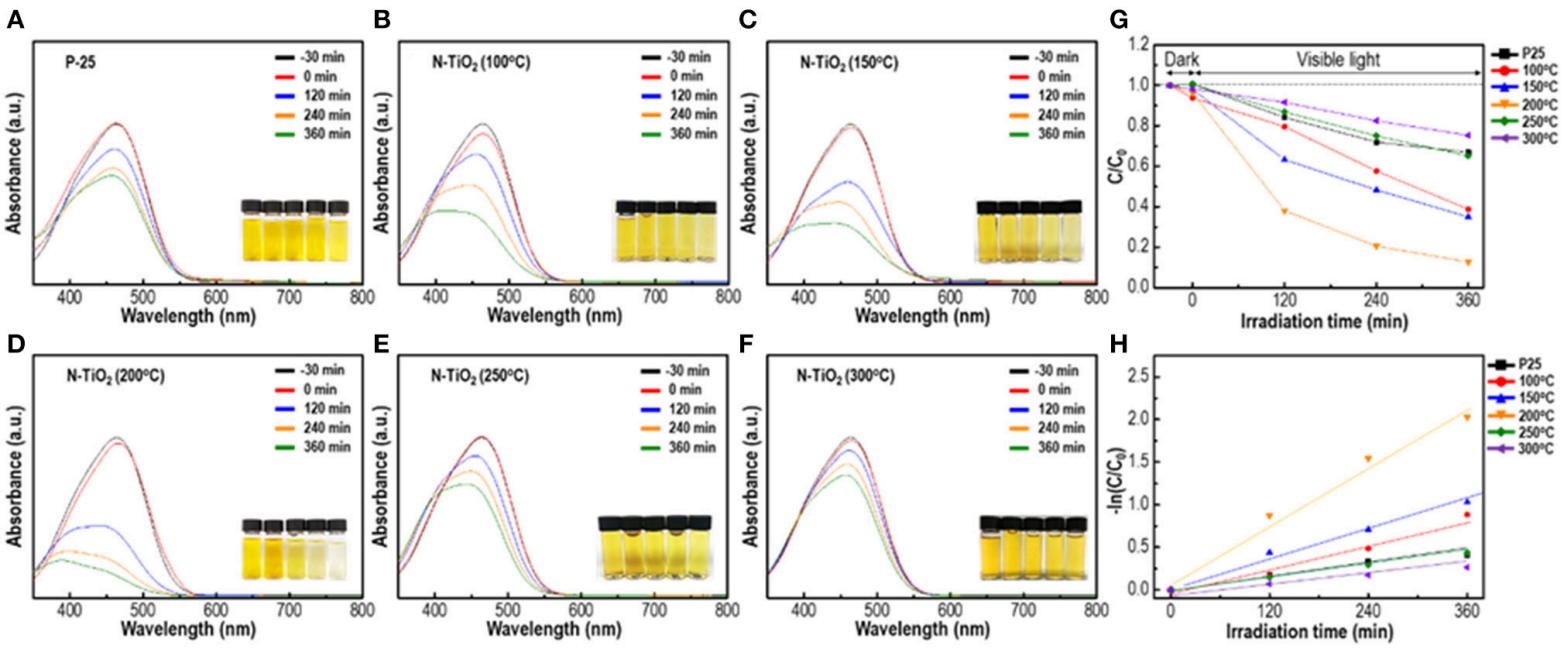
Photocatalytic activity of N-TiO_2_ within the visible region depending on different calcination temperature: **(A)** P-25, **(B)** 100°C, **(C)** 150°C, **(D)**200°C, **(E)** 250°C, **(F)** 300°C, **(G)** Photocatalytic efficiency, and **(H)** the plot of-In(C_0_/C).

To observe and compare photocatalytic activity depending on the calcination temperature and N doping content ratio, samples were mixed with a 1 × 10^−5^ MO solution and the degradation efficiency was analyzed. In Figures 7A–H, the as-prepared solution was irradiated with visible light (>400 nm) and the photocatalytic decomposition process was initiated. Degussa P-25 in Figure 7A, a standard TiO_2_ nanopowder, exhibited a reduced degradation activity of the MO solution, which was only 21% under visible region irradiation. The calcined samples in Figures 7B–F at 100–300°C of the anatase phase with various degrees of N doping and crystallinity exhibited an enhanced maximum efficiency activity of 91% that was 4 times greater than the degradation performance of P-25 within a reaction period of 6 h. The 200°C calcined N-TiO_2_ nanoparticles in Figure 7D yielded the greatest photodegradation results and recyclability was measured by cycling test in Figure S2. These results indicated that the anatase structure with 0.4 at.% N doping performed much better than the P-25 standard nanoparticles; the other calcination temperature samples yielded somewhat unpredictable phenomena, which should be discussed with regard to crystallinity and N doping content. The band gap of TiO_2_ generally depended on the electron state of the doping species and content. Among those, the N doping effect was widely accepted; in the TiO_2_ crystalline lattice, N incorporated and modified the electronic band structure, forming an N 2p band above the original O 2p valance band leading to the formation of a narrower band gap of minimum 2.5 eV and shifting the absorption region toward that of visible light. Undoped anatase TiO_2_ structure has larger band gap of 3.2 eV, which cannot be excited with visible light. On the other hand, when the N-doped TiO_2_ was exposed in the visible region, valence band at N 2p state above original O 2p reduces band gap and generates electrons in the conduction band while remaining holes in the valence band. The generated electrons on conduction band captures oxygen molecules in solution which produces highly reactive superoxide anions and hydroxyl radicals on the surface while holes in the valence band produces highly oxidative hydroxyl radicals by reacting with OH^−^ ions (Ansari et al., 2016). Not only N doping but also the Ti^3+^ donor contributed toward the absorption of light. Under visible light irradiation, nanoparticles excited and produced electron-hole pairs, which diffused into the particle surface. These electrons reduced Ti^4+^ into Ti^3+^ species that existed under the conduction band unless they were not trapped (Yang et al., 2010). However, prior studies proposed and certified that the N state above the valance band and Ti^3+^ that was produced by Ti^4+^ capturing electrons on the 1.2 eV conduction band that narrowed the band gap state could act as electron traps near the oxygen vacancy sites, reducing the electron-hole recombination duration time. Another parameter that affected the photodegradation efficiency was the crystallinity of the nanoparticles. It was already known that an amorphous-like layer of nanoparticles would occur due to its adsorption of water molecules on the particle surface. This would reduce the catalytic performance by masking interactions between particles and dye molecules. By removing the adsorbed species and increasing crystallinity, the photocatalytic properties could be enhanced (Yang et al., 2011). Thus, the conditions to obtain the highest photocatalytic properties within the visible light region should be optimized by adjusting the degree of crystallinity and N doping content (Jang et al., 2001; Zhoa et al., 2008; Wang et al., 2009). Following this approach, N-TiO_2_ calcined at 100°C with a doping content of 0.52 at.% could be applied as a trapping agent; the photodegradation efficiency was reduced compared to the 200°C condition. Additionally, at lower calcination temperatures, physically embedded or adsorbed water molecules and applied electrolytes on the surface were crucial factors that limited active sites, motivating the reduction in degradation efficiency. At conditions >250°C, negligible N doping content was observed due to an unstable state amid a highly crystalline structure; the poor degradation properties compared to P25 indicated that N doping was passivated by oxidation and returning the material to its original anatase structure while widening the band gap, which would limit its contribution toward enhancement of the visible light region catalyst.

### Photoluminescence analysis from various calcination temperatures

**Figure 8:**
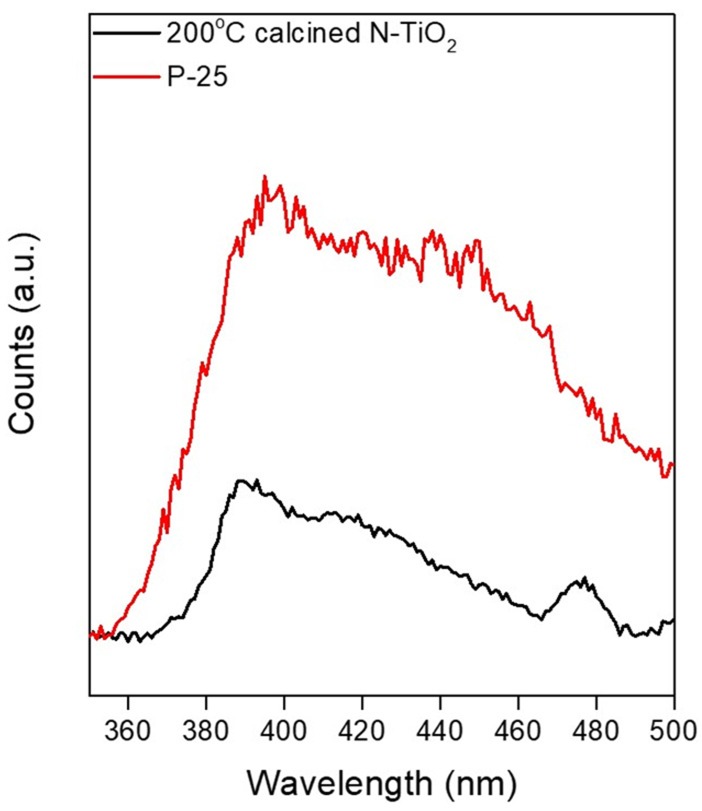
PL spectra of P-25 TiO_2_ and 200°C calcined 0.4 at.% N-TiO_2_.

To evaluate the radiative recombination of excited electrons and holes, synthesized N-TiO_2_ with 200°C calcined samples were analyzed via PL spectra under an excitation wavelength of 290 nm and compared to an undoped P-25 TiO_2_ powder reference in Figure 8. Excited peaks in the spectra were observed at 390, 424, 441, and 452 nm indicating different emission sources. The peak at 390 nm could be attributed to the conduction to valence band energy state transition, peaks at 424 and 452 nm were signals from free excitations at the band edge and the excitation peak at 441 nm was due to localized self-trapped excitons (Jagadale et al., 2008; Kho et al., 2010; Ablat et al., 2014). In addition to the PL analysis, the intensity virtually decreased for the 200°C calcined sample, showing that the lifetimes of excited electrons and holes with regard to the N doped TiO_2_ nanoparticles were greatly enhanced and contributed to decomposition reactions with organics until the electrons and holes recombined. These results coincided with the photocatalytic activity and XPS analysis showing that for temperatures >250°C, the N doping content was eliminated and the band gap recovered to its original value. Thus, photocatalytic activity would only occur within the UV region.

### Growth mechanism of N-TiO_2_ nanoparticles via plasma enhanced electrolysis

**Figure 9:**
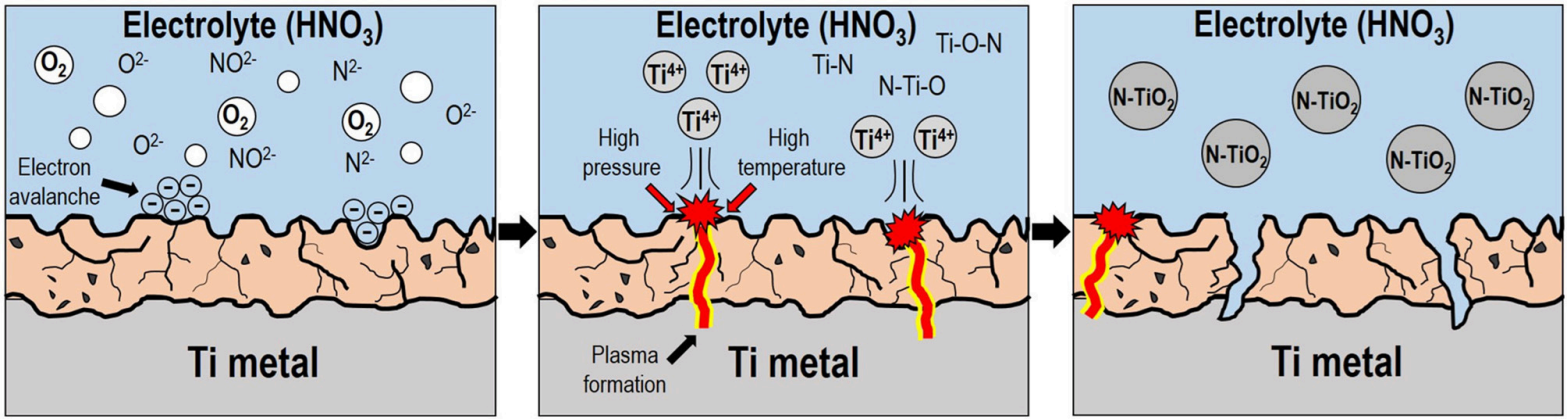
Schematic diagram of the N-TiO_2_ nanoparticle synthetic mechanism via plasma enhanced electrolysis.

To clarify the growth mechanism of the N-TiO_2_ nanoparticles using plasma assisted electrolysis, a schematic diagram can be seen in Figure 9 with the following description: (1) During the initial stage, low voltages produced a porous thin TiO_2_ oxide layer due to anodization during the pre-spark treatment period. (2) This increased the resistivity between the anode-electrolyte, forming an electric field strong enough to liberate and form as gas enveloped the anode surface and initially increased the applied voltage. (3) High voltages greater than the breakdown voltage at the electron avalanche birthplace excited the electrolyte near the anode via ionization and dissociation, transforming gases into ions such as NO^2−^, N^2−^, and O^2−^, which were subsequently localized onto the anode surface by applying considerable kinetic energy (Mikheev et al., 2015). (4) The ionized gaseous layer provided sufficient energy to initiate Joule heating, which entailed high temperatures and pressures, forming a plasma discharge within the gas envelope. Such arc plasma created Ti^4+^ metal cations from the outer surface of the anode into the electrolyte solution while the plasma gradually penetrated the inner part of the anode. (5) The metal cations then reacted with the as-prepared anionic gaseous species; the ionized cations initially reacted with N species from the ionization of nitric acid, a doping source of nitrogen, forming N-Ti-O bond structures within the Ti lattice through nucleation and growth mechanism; N-TiO_2_ nanoparticles were subsequently produced and assisted by controlled temperature induced cooling processes (Cheng et al., 2012). Moreover, the scarcity of existing anions in solution could be attributed to the repression of particle formation; acid-based nanoparticle synthesis conditions would prevent agglomeration between particles.

## Conclusions

Through the adoption of a novel synthetic method for amorphous N doped TiO_2_ nanoparticles using plasma enhanced electrolysis, massive N-doped TiO_2_ nanoparticles could be produced by merely supplying sufficient voltage to the anode, which was a metal source. This was because the synthetic phenomenon of the metal oxide nanoparticles occurred simultaneously via plasma generation with a rapid reaction.

Arc plasma formation was performed by applying a 500 V AC electric field to the nitric acid electrolyte. The particle morphology and size was verified from 12.1 to 24.7 nm relying on various nitric acid concentrations between the range of 5–15 mmol influenced by repulsive forces during nucleation. The N doping content and N-TiO_2_ nanoparticle crystallinity could be controlled by varying the calcination conditions and completely transforming the amorphous structure to anatase, enabling the control of N doping content from 0.54 to 0.4 at.%. N-TiO_2_ nanoparticles started to be synthesized just after applying voltage to the anode due to plasma generation, entailing a high temperature and pressure. The N-TiO_2_ nanoparticles synthesized after calcination at 200°C featured 0.4 at.% of N doping content and exhibited the greatest MO degradation performance of 91% in the visible region, which could be attributed to a narrow band gap structure due to N doping; PL analysis also revealed the most delayed recombination rate as well (see Figures 6, 7). Thus, this one-step metal-direct synthetic method via plasma generation demonstrated great potential for the novel and high-quality synthesis of N-doped metal oxide nanoparticles and could be scaled-up for industrial applications.

## Author contributions

TK prepared in writing this paper work through experiment and data analysis. G-MG helped measuring photocatalytic activities of nanoparticles. H-BC helped and advised result of this paper and modified it as logical, YS helped searching for the related information about this paper. C-GL and Y-HC equally contributed to this manuscript and accepted responsibility for conduct of research and final approval.

### Conflict of interest statement

The authors declare that the research was conducted in the absence of any commercial or financial relationships that could be construed as a potential conflict of interest.
