# Supplementary figures and images for: A Novel Synthetic Method for N Doped TiO2 Nanoparticles Through Plasma-Assisted Electrolysis and Photocatalytic Activity in the Visible Region
Source: Front Chem. 2018 Oct 2;6:458. doi: 10.3389/fchem.2018.00458 (PMC6183595; doi:10.3389/fchem.2018.00458)

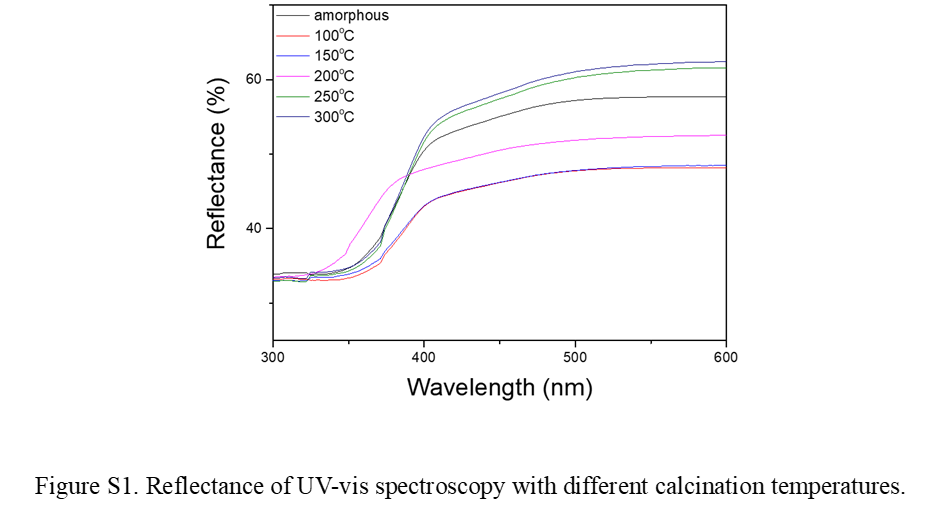

Supplement: Supplementary file 1 [file Image_1.TIF]

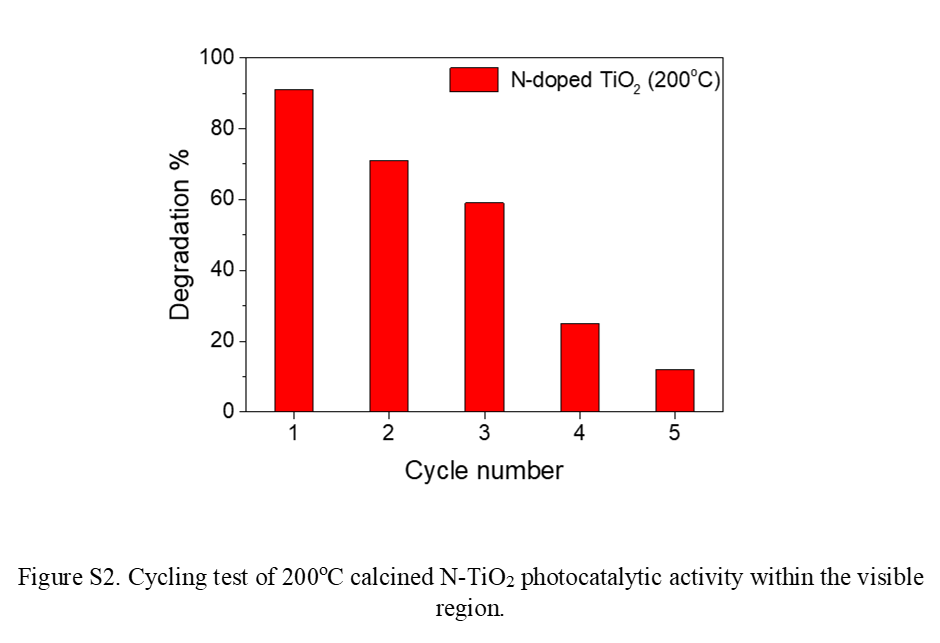

Supplement: Supplementary file 2 [file Image_2.TIF]
